# An electronic application for rapidly calculating Charlson comorbidity score

**DOI:** 10.1186/1471-2407-4-94

**Published:** 2004-12-20

**Authors:** William H Hall, Ramanathan Ramachandran, Samir Narayan, Ashesh B Jani, Srinivasan Vijayakumar

**Affiliations:** 1 University of California, Davis, Department of Radiation Oncology, UC Davis Cancer Center, 4501 X Street, G126, Sacramento, CA 95817 USA; 2 University of Chicago, Department of Radiation and Cellular Oncology, 5758 S Maryland Ave, MC 9006, Chicago, IL 60637, USA

## Abstract

**Background:**

Uncertainty regarding comorbid illness, and ability to tolerate aggressive therapy has led to minimal enrollment of elderly cancer patients into clinical trials and often substandard treatment. Increasingly, comorbid illness scales have proven useful in identifying subgroups of elderly patients who are more likely to tolerate and benefit from aggressive therapy. Unfortunately, the use of such scales has yet to be widely integrated into either clinical practice or clinical trials research.

**Methods:**

This article reviews evidence for the validity of the Charlson Comorbidity Index (CCI) in oncology and provides a Microsoft Excel (MS Excel) Macro for the rapid and accurate calculation of CCI score. The interaction of comorbidity and malignant disease and the validation of the Charlson Index in oncology are discussed.

**Results:**

The CCI score is based on one year mortality data from internal medicine patients admitted to an inpatient setting and is the most widely used comorbidity index in oncology. An MS Excel Macro file was constructed for calculating the CCI score using Microsoft Visual Basic. The Macro is provided for download and dissemination.

The CCI has been widely used and validated throughout the oncology literature and has demonstrated utility for most major cancers. The MS Excel CCI Macro provides a rapid method for calculating CCI score with or without age adjustments. The calculator removes difficulty in score calculation as a limitation for integration of the CCI into clinical research. The simple nature of the MS Excel CCI Macro and the CCI itself makes it ideal for integration into emerging electronic medical records systems.

**Conclusions:**

The increasing elderly population and concurrent increase in oncologic disease has made understanding the interaction between age and comorbid illness on life expectancy increasingly important. The MS Excel CCI Macro provides a means of increasing the use of the CCI scale in clinical research with the ultimate goal of improving determination of optimal treatments for elderly cancer patients.

## Background

Comorbid illness plays an essential, but poorly defined, role in the diagnosis and management of malignant disease. Increasingly, the importance of measuring comorbidity in consistent and quantifiable ways is being recognized. This movement has stemmed in part from a growing consensus that comorbidity confounds the results of clinical trials and limits the generalization of results to older and sicker patients [1,2]. For various reasons, however, the widespread integration of comorbidity into clinical research has yet to be realized. It is our contention that limited accessibility and cumbersome scoring techniques are in part responsible for the limited use of comorbidity indices. We believe that easily accessible tools for calculating comorbidity can increase their use in clinical research.

While multiple comorbidity indices are available, each with unique advantages and disadvantages, no single index has emerged as clearly superior to the others. In fact, we have noted that a distinct trade-off between prognostic utility and ease of use exists. We believe that a scoring system that maximizes ease of use while maintaining prognostic validity represents the optimal balance required for use in clinical research. In addition, a scoring system that can be easily integrated into an electronic medical record will further promote the widespread use of comorbidity data. We have, therefore, chosen to explore the use of the Charlson Comorbidity Index (CCI) as the prototypical comorbidity index in our department. The primary aim of this article, therefore, is to provide an electronic Charlson Comorbidity Index Scoring program and explain its development and use. In an effort to provide the reader with the context in which the electronic application was developed and should be used, the interaction of comorbidity and malignant disease and the validation of the Charlson Index in oncology are discussed. For more detailed reviews of the comorbidity indices outlined in this article, the authors refer readers to three systematic reviews of comorbid illness scoring systems by Extermann and de Groot [2-4].

### Comorbidity and cancer

In the 1960's, Feinstein initially reported the prognostic importance of patient-related characteristics, such as symptomatology and concurrent illness, in his analyses of differences between actual survival outcomes and those predicted by TNM-based staging among lung cancer patients [5]. In recent years, the direct influence of comorbid illness on treatment decision-making and survival outcomes has been documented for a variety of malignancies including bladder, lung, head and neck, colorectal, breast, and prostate cancers [6-11].

Hall, et al, for example, evaluated the effect of comorbidity on survival among head and neck cancer patients, concluding that 16% of mortality at 3 years and 18% at 5 years was attributable to comorbid illness alone, with non-cancer causes of death exceeding cancer-related causes of death after 7.5 years [6]. Satariano & Ragland made comparable observations in their study of breast cancer patients. In their analysis, comorbidity increased directly with age (p < 0.001) and a significant association between comorbidity and the type of treatment received (p < 0.0001) was observed. After controlling for age, cancer stage, and type of treatment, increasing comorbidity remained significantly predictive of increased all-cause-mortality (1 condition, p = 0.04; and for 2 or 3 conditions, p < 0.001) [7].

Comorbidity has also demonstrated marked predictive power for survival and treatment allocation among prostate cancer patients. A Netherlands cancer registry study, for example, identified comorbidity as the single most important prognostic factor for 3-year survival, with hazard ratios of 2.0 (95% CI = 1.0–4.3) for a single comorbid illness and 7.2 (95% CI = 3.1–16.6) for 2 or more comorbid conditions, and trends toward fewer radical prostatectomies among men with higher degrees of comorbidity [8]. Total comorbidity counts have been found to be strongly predictive of survival among colon cancer patients as well. In addition to identifying increasing comorbidity with age (p < 0.0001), Yancik, et al, found raw counts of comorbid conditions to be strongly predictive of survival when used in a model containing age group, disease stage, and gender (p = 0.0007), with risk ratios of 1.11 (95% CI 1.10–1.90) and 1.84 (95% CI 1.39–2.46) for total comorbid illness counts of 5–6 and 7–14 respectively [9]. Additional works by De Marco with colon cancer patients [10], Firat with lung cancer patients [11], and Piccirillo with head and neck cancer patients [12] provide unquestionable support for the importance of comorbidity on survival and treatment-related complications among oncology patients.

Although the preceding examples are not intended to provide a comprehensive review of the influence of comorbidity on survival and treatment-related complications in oncology, they provide a clear demonstration of the effect. In addition, the themes of increasing comorbidity with age and the influence of comorbidity on outcomes and treatment decision-making are illustrated. With these interactions in mind, the investigation of comorbidity has become an area of increasing interest in our department. In particular, we have begun focusing on the use of comorbidity indices and their application in clinical research. For a variety of reasons, which will be explained in forthcoming sections of this work, we have focused on the Charlson Comorbidity Index as the prototypical index on which to base this research.

### The Charlson Comorbidity Index

The Charlson Index was developed in 1987 based on 1-year mortality data from internal medicine patients admitted to a single New York Hospital and was initially validated within a cohort of breast cancer patients. The index encompasses 19 medical conditions weighted 1–6 with total scores ranging from 0–37. In the development phase of the index, mortality for each disease was converted to a relative risk of death within 12 months. A weight was then assigned to each condition based on the relative risk (RR); for example, RR <1.2 = weight 0, RR ≥ 1.2<1.5 = weight 1, RR ≥ 1.5<2.5 = weight 2, RR ≥ 2.5<3.5 = weight 3, and for 2 conditions (metastatic solid tumor and AIDS) = weight 6.

From the weighted conditions, a sum score can be tallied to yield the total comorbidity score. The CCI can be further adapted to account for increasing age. In the validation phase of the CCI, age was also found to be an independent risk factor for death from a comorbid condition. As a result, relative risk was calculated to increase by 2.4 for each additional decade of life. In the same cohort, the relative risk of death for each 1-point increase in CCI score was 2.3. To account for the effects of increasing age, one point can be added to the CCI score for each decade of life over the age of 50 [13].

Reviews of the CCI suggests it has good reliability, excellent correlation with mortality and progression-free survival outcomes, and is easily modifiable, particularly to account for the effect of age. The CCI's basic limitations include preservation of data only for the 19 conditions listed in the index, the exclusion of non-malignant hematologic disease, such as anemia, and reduced predictive ability for outcomes < 6-months. The CCI is praised for its ease of use, short rating time, extractability from other indices, and widespread use [2,3].

### Validation of the Charlson Index

Statistical criteria for the assessment of the validity of a test include content validity, criterion validity, construct validity, and reliability [4]. Although a detailed discussion of statistical tests of validity is beyond the scope of this review, the assessments provide a basis from which to begin an analysis of the validity of the Charlson Index. Statistical criteria of validity, as applied to comorbidity indices, are ultimately dependent upon the comparison of comorbidity indices to each other, as well as subjective assessments of certain criteria, such as content validity and cutoff points for correlation coefficients. The criteria are, therefore, in and of themselves, problematic. Despite these limitations, their application to the common comorbidity indices has been studied extensively.

In a review of validity among comorbidity indices, de Groot, et al, systemically identified articles referring to comorbidity between 1966 and 2000. They compared the Charlson Index with the Cumulative Illness Rating Scale (CIRS), Kaplan-Feinstein Index (KFI), and Index of Coexistent Disease (ICED) and identified correlation coefficients of > 0.40, "good" test-retest reliability and "moderate to good" inter-rater reliability for the CCI [4]. In addition, the Charlson Index correlated significantly with mortality, disability, readmission, and length of stay outcomes, suggesting good predictive validity leading de Groot, et al, to conclude that the Charlson Index, as well as the ICED, KFI, and CIRS, is a valid and reliable method for assessing comorbidity in clinical research [4].

A similar review by Extermann suggests the Charlson Index possesses excellent validity and reliability for use in clinical research in oncology. Extermann also reported exceptional predictive validity, correlating the CCI with outcomes involving mortality risk from weeks to years, postoperative complications, length of hospital stay, discharge to nursing home, and progression-free survival among cancer patients. Additionally, inter-rater reliability, by various measures, was reported at 0.74 among a cohort of older general oncology patients and 0.945 within a group of elderly breast cancer patients. Test-retest reliability was also good, ranging from 0.92 among surgical patients and 0.86 among the previously mentioned group of elderly oncology patients. Although Extermann urges some caution based on the tendency of the CCI to result in comorbidity scores that are sometimes lower than those observed with other indices, she concludes that the CCI is easy to use and "highly suitable for vast cohort studies but may under-detect significant problems resulting in non-lethal endpoints" [2].

The Charlson Index has demonstrated excellent predictive validity for a variety of clinical outcomes as well as numerous malignancies. As discussed previously, the CCI was developed using a prospective analysis of 1-year mortality rates among internal medicine patients and then validated within a population of 588 breast cancer patients. In the validation phase of Charlson's original study, increasing CCI scores were significantly correlated with increased 10-year mortality within a breast cancer cohort (χ^2 ^= 163, p < 0.0001), with CCI scores of 0, 1, 2, and 3 predicting 10-year survival rates of 93%, 73%, 52%, and 45%, respectively. In the original manuscript, Charlson, et al, cautioned that their index should be considered preliminary and that it required validation in larger populations [13].

Since the original work by Charlson, et al, the CCI has exhibited substantial prognostic power for both survival and treatment related complications in numerous retrospective studies. Singh, et al, for example, retrospectively analyzed CCI validity within a cohort of head and neck cancer patients. Their analysis revealed reduced median tumor specific survival (12.3 vs. 38.7 months, p = 0.007), and increased risk of cancer death (RR = 2.35) for patients with advanced (≥ 2) CCI scores. The CCI compared similarly to the KFI with respect to frequency of advanced comorbidity (30% for CCI and 32% for KFI) and prognostic power (Spearman correlation coefficient, p <0.001, r = 0.73). However, the CCI was more applicable to the study population than the KFI, with the KFI successfully applied to only 80% of the study population compared with 100% application of the CCI [14].

Fowler, et al, also examined the validity of the Charlson index in a cohort of men with prostate cancer treated with EBRT or RP. After adjusting for age, a direct relationship between actuarial survival and CCI score (p = 0.00001) was found for all patients. Among individuals with CCI scores of 0, 5 and 10-year survival rates were 86% and 66% compared with 40% and 9% for patients with CCI scores of 3 to 5. Relative mortality risk, based on CCI scores of 0, 1, 2, and 3–5, increased from 1 to 1.7, 2.6, and 5.7, respectively [15].

Additional studies among prostate cancer patients have compared the CCI, KFI, and ICED. Albertsen, et al, for example, found each of the three comorbidity indices had similar power to predict survival (p < 0.001 for each), with the addition of any of the three indices to Gleason score improving predictive power for survival over Gleason score alone [16]. We also recently reviewed the importance of comorbidity and prognostic utility of the CCI among prostate cancer patients and found that the CCI consistently correlates with reduced survival as well as treatment allocation [17].

The Charlson Index has also been validated as a prognostic indicator for survival in lung cancer cohorts. Firat, et al, recently explored the prognostic importance of comorbidity among patients undergoing surgical resection or definitive EBRT for clinical NSCLC. Within the combined group, both CIRS-G scores ≥ 4 (p < 0.001) and Charlson score ≥ 2 (p = 0.004) emerged as significant prognostic indicators of reduced overall survival. Examination of the surgical and EBRT groups separately also demonstrated higher CIRS-G and Charlson scores within the EBRT group as compared with the surgical group [18].

The effect of comorbidity on complication rates among lung cancer patients has also been investigated. Brim, et al, for example, identified gender, CCI score 3–4, COPD, and prior tumor within the last 5 years as predictors for major complications (re-thoracotomy, empyema, pleural effusion, bronchopleural fistula, ventilatory support >72 hours, ventricular arrhythmia, pulmonary embolism, cardiac failure, or myocardial infarction). Charlson scores of 3–4 maintained statistical significance after multivariate regression (OR 9.8, 95% CI 2.1–45.9) [19].

CCI scores have also demonstrated prognostic value, both in terms of postoperative complications and survival among colon cancer patients. Rieker, et al, found raw CCI scores reached 0–2, 3–4, and ≥ 5 in 66%, 25%, and 8% of patients, respectively. With respect to survival, CCI score >2 emerged as a poor prognostic indicator for overall survival for all stages (p < 0.001, OR 2.91, 95% CI = 2.00–4.94). Subgroup analysis of stage III and IV patients revealed reduced cancer-specific survival among patients with CCI score >2 (log rank p <0.005). CCI scores > 2 were also correlated with receipt of blood transfusion (p < 0.021, OR 1.56, 95% CI = 1.07–2.28), postoperative complications (p < 0.001, OR 2.18, 95% CI = 1.50–3.16), and ICU stay > 2 days (p < 0.001, OR 3.28, 95% CI = 1.91–5.64) [20].

Taken together, this series of papers represents a diverse and relatively large experience with the Charlson Index. In each report, CCI scores consistently correlate with disease specific survival, overall survival, or treatment-related complications, confirming its predictive validity.

## Implementation

The CCI Calculator provided with this manuscript is based on the original index proposed by Charlson, et al, and is available in the section: supplementary material/table 1/appendix 1 [see additional file: CCICalc.xls]. The calculator was developed using Microsoft Excel/Visual Basic software and can be downloaded from this journal. Simplicity and ease of use were the main design objectives.

Presented as a simple Microsoft Excel tool, it can be easily extended or integrated with other systems that can import Microsoft Excel data, or imported as a flat file. The Calculator functions well with both MS Windows and Macintosh operating systems running any Microsoft Excel version with Macro capabilities and is free to all users of Biomed Central Cancer. There are no restrictions concerning the use of the calculator software. A running CCI score can be calculated by selecting the conditions and age groups within the file. The calculator can be used with or without age modification as proposed by Charlson, et al [13]. It is important to note that the upper limit scores for this calculator are 37 for "age unadjusted" and 43 for "age adjusted." Charlson scores >8–10 have not received extensive evaluation in the comorbidity literature. We intend the calculator to be widely distributed so that use of the CCI can become a routine aspect of clinical research in oncology.

To use the calculator, the user must select "enable macros" when prompted to do so as the file opens. To calculate a CCI score, any of the applicable conditions can be selected. All selected conditions will then be displayed in a lighter shade within the table. Corrections can be made by deselecting conditions, which then removes their weighted value from the score. The CCI score can then be totaled, or an age-modified score can be determined by selecting any one of the applicable "Age by Decade" groups. Scores totaled without age modification will appear in the "Age Unadjusted CCI Score" total and no value will appear in the "Age Adjusted Score" total. Scores totaled by selecting an age group without selecting a comorbidity will result in no value for either total and the user will be prompted to "Reset & Select Condition." To reset the program, the "Reset CCI Calculator" button can be selected. The calculator can be further modified as needed by changing entries in the "Data Sheet" area of the workbook which is hidden in the read-only version of the calculator, but can be unhidden by selecting "Format," then "Sheet," followed by "Unhide" from the Excel menu. The "Data Sheet" can then be selected and will be viewable. To modify the original Macro, users can contact the authors and the password will be provided on a case-by-case basis.

## Results and discussion

The extensive validation of the CCI as a powerful predictor of clinical outcome combined with its simplicity and widespread use in oncology have led to the adoption of the Charlson Index as the prototypical comorbidity index in our department. In addition to validity, our criteria for the use of a comorbidity index focus on simplicity in design, consistency in scoring, and ease of use. It is our contention that many of the commonly used comorbidity indices, such as the ICED, CIRS, and KFI have failed to achieve widespread use because they remain complicated, cumbersome to use, and poorly accessible for use in clinical research. Given the adaptability of the CCI for the inclusion of additional variables, such as age, the CCI also demonstrates marked potential for modification into cancer specific comorbidity indices. We have, therefore, developed a Charlson Comorbidity Calculator based on a Microscoft Excel File to improve the collection of comorbidity data in our department.

Comorbid illness has demonstrated increasing importance as a prognostic factor for survival and treatment-related outcomes in oncology. It confounds the results of clinical trials because the lack of a standardized measurement has resulted in the failure to adjust for comorbidity in statistical analysis of outcomes data [1,2]. It also limits the applicability of clinical research to large segments of the oncology population because protocol designs tend to exclude older and sicker patients [21,22]. Recent reviews consistently identify the CCI, ICED, CRIS and KFI as validated and acceptable measurements of comorbidity and recommend their use in clinical research. Although the ICED, CIRS, and KFI obtain superior prognostic power in some series, the CCI consistently demonstrates statistical validity, particularly in terms of prognostic validity, and remains the most structurally simple, easy to use and well-defined of the comorbidity indices. The ICED and CIRS, for example, both require coding manuals and training courses to be used effectively. The KFI has required extensive modification for use in oncology because it was originally designed to assess comorbidity in diabetic patients. Recent modifications of the KFI for use in oncology, such as those applied by Piccirillo in a head and neck cancer specific modification of the KFI (available in electronic calculator format at ) also require training courses for effective use [12]. By contrast, the Charlson Index is intuitive, requiring users to select a condition from a defined list, rather than searching for disease value or specific information about disease severity. In our department, the cumbersome requirements for use of the ICED, KFI, and CIRS would reduce compliance with collection of comorbidity data. Furthermore, the increased training requirements and intricacies of these indices may increase variability between scores, as it is unlikely that a single staff member would be responsible for the collection of all data. It is, therefore, our belief that the Charlson Index represents the optimal balance between ease of use and prognostic ability and has, therefore, become the method of choice for the collection of comorbidity data in our department. Accordingly, we developed the CCI calculator to improve compliance with the collection of comorbidity data and as a quality assurance tool to ensure that such data is collected correctly and uniformly.

The use of comorbidity data in clinical research is at an important crossroads, with necessity of its use becoming imperative as electronic capabilities for its assessment become more feasible. As the US population gets older, the use of comorbidity data in clinical trials will only increase in relevance. Current estimates indicate that the elderly will comprise 20% of the population by the year 2030 [23]. Studies of older oncology patients also suggest that the elderly shoulder the majority of cancer burden, with risk rates 11 times greater than those of younger patients, with over 50% of all cancer-related mortality [24]. The rise of comorbidity with increasing age is a theme common to most retrospective studies of comorbidity. In this light, determining the effect of comorbidity on cancer-related survival and treatment-related complications has become increasingly important.

Furthermore, evidence to suggest that comorbidity and performance status represent independent prognostic factors is accumulating. Extermann, et al, for example, examined the relationship between comorbidity and performance status. Both Charlson and CIRS-G were found to have little or no correlation with ECOG performance status, activities of daily living (ADL), or instrumental activities of daily living (IADL). More recently, Repetto, et al, found that among 269 elderly cancer patients with a reported ECOG performance score of <2, 13% had 2 or more comorbidities, 9.3% had ADL limitations, and 37.7% had IADL limitations. Although a statistical correlation between ECOG performance status, number of comorbidities, and comprehensive geriatric assessment was identified in univariate analysis, only comorbidity, ADL limitation and IADL limitation maintained statistical significance in multivariate analysis. Firat, et al also found CIRS-G and Karnofsy performace status to be independent predictors of outcome in their analysis of prognostic factors in 112 patients enrolled on 4 RTOG trials of stage III lung cancer [11].

Without widespread integration of comorbidity data into clinical research, an increasing number of elderly patients, and their physicians, will be left with treatment recommendations and outcomes data that lack relevance for their age and level of comorbidity.

Concurrently, electronic medical records (EMR) and data collection systems are becoming increasingly common and easy to use, with EMR use among European countries approaching 60% to 90% [27]. The EMR ultimately promises increased physician efficiency and improved clinical outcomes for patients. Contemporary EMR systems have improved outcomes by reducing errors with the use of electronic prescribing systems and improving preventative care with automated reminder systems [28,29]. The MS Excel CCI Calculator provided with this manuscript, for example, could easily be integrated into an EMR for aid in data collection. Such integration would eventually provide an enormous data pool on which to base future research on the prognostic importance of CCI.

To our knowledge, this is the first electronic data collection system offered for the Charlson Comorbidity Index. The simplicity of the index itself, coupled with the simplicity of MS Excel and the Visual Basic programming language, have resulted in a robust electronic CCI calculator that functions well across both Windows and Macintosh platforms. The latest version of the calculator, which is provided with this manuscript, has performed without error consistently on the first (WH), second (RR) and third (SN) authors' Windows-based PCs.

The major limitations of the CCI calculator lie in the limitations known to comorbidity indices and to the index itself. These include lack of understanding as to the relative importance of various individual conditions on mortality, treatment-related complications and quality of life. Furthermore, failure to include some conditions with particular relevance to cancer patients, such as non-malignant hematopoietic disorders and thromboembolic disorders, as well as uncertainty as to whether a few specific diseases or the overall disease burden is more important for prognosis, remain important considerations limiting use of the CCI [2,3]. Additionally, the CCI has a tendency to underscore comorbidity because it is limited to 19 conditions and because it excludes the primary malignant condition. For example, in a patient with localized prostate cancer, history of COPD and myocardial infarction, the CCI score calculated by a urologist would exclude prostate cancer from the calculation resulting in a score of 2. The same patient might receive a score of 3 by a cardiologist because myocardial infarction, as opposed to prostate cancer, was excluded from the calculation. Another limitation of the CCI lays in the frequent use of grouped CCI scores, or CCI grades, rather than the use of scores as continuous variables. Within an elderly cohort in whom comorbidity is likely to be high, the CCI will have reduced utility if it lacks the ability to distinguish between a score of 2, representing mild to moderate comorbidity, and a score of 8, representing severe comorbidity. With this limitation in mind, we recommend the use of CCI score as a continuous variable.

Despite its limitations, the general oncology literature supports the use of CCI as a prognostic variable in clinical research. It should be emphasized that the CCI is not meant to replace clinical experience and its use in clinical decision-making should be considered investigational. With additional research, CCI methodological limitations can be addressed and the index modified to improve upon its utility. In an effort to improve our understanding of the CCI and identify areas of the index in need of improvement, we are currently investigating the effect of score thresholds on treatment decision-making among prostate cancer experts. We believe that dissemination of the MS Excel CCI Macro will lead to increased use of the CCI for clinical research purposes as well as modification of the CCI to increase its validity and clinical utility. Ultimately, we hope that the comorbidity indices, such as the CCI, will see widespread use in clinical research and eventual integration into EMRs as a result of these efforts.

## Conclusions

The Charlson Comorbidity Index has demonstrated excellent predictive validity in numerous cancer-related outcome studies. It has met the criteria for statistical validity as outlined by several authors. In our opinion, the CCI represents the optimal balance between ease of use and prognostic ability. Its simplicity in design also makes its adaptation to include additional variables extremely feasible. We have, therefore, adopted the CCI as an acceptable comorbidity measurement tool in our department and created a Microsoft Excel Macro to facilitate its correct and uniform use in clinical research.

## Availability and requirements

• **Project name**: Charlson Comorbidity Calculator

• **Project home page**: None

• **Operating system(s)**: Windows or Macintosh OS

• **Programming language**: Visual Basic

• **Other requirements**: Microsoft Excel (macro enabled)

• **License**: None

• **Any restrictions to use by non-academics**: None

## List of abbreviations

• **CCI**: Charlson Comorbidity Index

• **ICED**: Index of Co-Existent Disease

• **KFI**: Kaplan-Feinstein Index

• **CIRS**: Cumulative Illness Rating Scale

• **RR**: Relative Risk

• **EMR**: Electronic Medical Record

• **CDSS**: Computer-Based Decision Support Services

## Competing interests

The author(s) declare that they have no competing interests.

## Authors' contributions

WH, SN, AJ, and SV carried out the literature review, assembly and editing of the manuscript. RR created the CCI calculator. All authors read and approved the final manuscript.

## Pre-publication history

The pre-publication history for this paper can be accessed here:



## Supplementary Material

Additional File 1 A Microsoft Excel (CCICalc.xls) is included with this manuscript and can be found in supplementary material/table 1/appedix 1. A detailed description of the creation of the file and instructions for its use are included in the implementation section of this manuscript. To Calculate a Charlson Comorbidity Index score using the calculator double click on the CCI-Calc.xls icon or open the file from MS Excel. You must select "enable macros" when prompted to do so by the MS Excel macro warning pop-up window. A CCI score can then be calculated by selecting the conditions and age groups within the file. Selected conditions will appear in the table as a lighter shade than deselected conditions. As comorbidities are selected a running total of the score will be calculated. Scores totaled without age modification will appear in the "Age Unadjusted CCI Score" total and no value will appear in the "Age Adjusted Score" total. A selected condition can be deselected by clicking on once on the button for that condition. A score may be calculated without selecting an age category, however Scores totaled by selecting an age group without selecting a comorbidity will result in no value for either total and the user will be prompted to "Reset & Select Condition." Once finished with a calculation, the calculator can be reset by selecting the green "Reset CCI Calculator" button. The file is presented in a password protected format so that no changes can be made to the categories and weighting as proposed in the original Charlson Comorbidity Index. Click here for file
